# Pregnant Women’s Attitudes and Beliefs towards Sleep and Exercise: A Cross-Sectional Survey

**DOI:** 10.3390/clockssleep5010004

**Published:** 2023-01-17

**Authors:** Summer Cannon, Melanie Hayman, Michele Lastella

**Affiliations:** 1 School of Health, Medical and Applied Sciences, CQUniversity, Rockhampton, QLD 4701, Australia; 2 Appleton Institute for Behavioural Science, CQUniversity, Adelaide, SA 5034, Australia

**Keywords:** pregnancy, sleep, exercise, attitudes, beliefs, barriers

## Abstract

As many as 80% of women report experiencing poor sleep throughout pregnancy. Exercise is associated with many health benefits during pregnancy and is established as a non-pharmacological method to improve sleep in both pregnant and non-pregnant populations. Given the importance of sleep and exercise during pregnancy, the aim of this cross-sectional study was to (1) examine pregnant women’s attitudes and beliefs towards sleep and exercise during pregnancy, and (2) investigate the barriers women face to achieving good sleep and engaging in healthy levels of exercise. Participants were comprised of 258 pregnant Australian women (31.3 ± 5.1 years) who completed a 51-question online survey. Almost all (98%) participants believed exercise during pregnancy to be safe, whilst over half (67%) believed participating in more exercise will improve their sleep. Over 70% of participants reported experiencing barriers such as physical symptoms related to pregnancy that negatively impacted their ability to exercise. Almost all (95%) participants reported experiencing barriers to sleep in their current pregnancy. Present findings suggest that overcoming intrapersonal barriers should be a priority for any intervention aiming to improve sleep or increase exercise levels in pregnant populations. Findings from the present study highlight the need for a better understanding of women’s sleep experiences during pregnancy, and demonstrate how exercise may improve sleep and health outcomes.

## 1. Introduction

It is recommended that adults, including pregnant women, obtain 7–9 h of sleep per night [1]. However, pregnant women experience less sleep, increased wakefulness, and excessive daytime sleepiness compared to non-pregnant populations [2], with 80% of women reporting poor sleep throughout pregnancy [3,4]. Poor sleep during pregnancy is associated with an increased risk of adverse maternal and fetal health outcomes including preeclampsia, gestational diabetes mellitus, caesarean delivery, and preterm birth as well as general tiredness, fatigue, and cognitive impairment [3,5,6,7]. The need for non-pharmacological approaches to improve poor sleep during pregnancy has been established due to the safety risks posed by pharmacological methods [8,9].

Exercise is a healthy, safe, and effective option for the management of poor sleep in non-pregnant populations [10]. Exercise includes but is not limited to recreational, transportation, and caregiving activities as well as structured exercise [11]. The positive psychological effects of exercise, in addition to its restorative functions, are thought to play a role in improving sleep [12]. Furthermore, there is evidence to suggest that sunlight exposure gained during outdoor exercise may work to improve circadian rhythm and thus, sleep [12,13,14]. During pregnancy, exercise is associated with several health benefits such as a reduced risk of gestational diabetes [15], preeclampsia, and the need for obstetric intervention (e.g., caesarean birth) [16,17]. Despite the well-established benefits of exercise during pregnancy, research suggests that most pregnant women are not meeting recommended levels of exercise during pregnancy [18,19,20]. Women report a wide range of barriers that prevent them from engaging in adequate amounts of exercise including physical symptoms and psychosocial factors [21]. The Australian guidelines for physical activity during pregnancy recommend pregnant women engage in 150–300 min of moderate- to vigorous-intensity activity per week, accumulated across most, if not all days of the week [18]. Attitudes and beliefs towards exercise are a significant factor regarding exercise participation among pregnant women [22,23]. Similarly, positive attitudes towards sleep are associated with longer sleep duration and improved sleep hygiene [24]. While a recent systematic review indicates that exercise improves sleep among pregnant women [25], little is known about pregnant women’s attitudes and beliefs towards the use of exercise as a means for improving sleep.

Given the adverse health outcomes associated with poor sleep and inactivity during pregnancy, the aim of this study was to (1) examine pregnant women’s attitudes and beliefs towards sleep and exercise during pregnancy, and (2) investigate the barriers women face to achieving good sleep and engaging in sufficient levels of exercise.

## 2. Results

A total of 258 pregnant Australian women aged between 17 and 42 years (31.3 ± 5.1 years) volunteered to complete the survey at their convenience. The majority of women were pregnant with their second baby, married, held a bachelor degree, and were employed full-time with an annual household income above $150,000 (Table 1).

### 2.1. Attitudes and Beliefs Surrounding Sleep during Pregnancy

Sixty-three percent (154/246) of participants felt they get less sleep now than before they were pregnant. Approximately 90% of participants reported experiencing occurrences of tiredness. Over 34% (86/246) reported feeling tired very often (22%) or always (13%), while a further 60% (157/246) of participants reported feeling tired sometimes (24%) or often (39%). When asked how often participants woke on average per night, responses ranged from 0 to 10. The most frequently reported number of wake times per night was three times (51/246). Participants were asked on average how many hours of sleep they believed they should get per night and answers ranged between 4–12 h with 8 h per night being the most frequently reported answer (132/246).

### 2.2. Attitudes and Beliefs towards Exercise

Women reported the following benefits to exercising during pregnancy: reduced anxiety and depression (77%, 50/65), improved emotional well-being (72%, 47/65), improved physical fitness (69%, 45/65), improved sleep quality (63%, 41/65), reduced back pain (60%, 39/65), reduced postpartum weight retention (57%, 37/65), control over weight gain (54%, 35/65), decreased risk of pregnancy complications (such as pregnancy-induced hypertension and preeclampsia (52%, 34/65), faster return to pre-pregnancy weight (52%, 34/65), reduced pelvic pain (41%, 27/65), fewer delivery complications (such as caesarean section (41%, 27/65), prevention and management of urinary incontinence (37%, 24/65), all of the above (35%, 23/65), there are no benefits (0%, 0/65), and other (1%, 1/65). Only 67 out of 258 (26%) women chose to disclose whether they are participating in exercise in their current pregnancy. Of those, 70% answered yes (47/67), and 30% (20/67) answered no.

### 2.3. Attitudes and Beliefs towards the Safety of Exercise during Pregnancy

Nearly all (98%, 250/254) participants reported believing exercise during pregnancy is safe. Participants were asked how many days per week they feel it is safe to exercise during pregnancy. Answers varied between 2 and 7 days with 7 days being the most frequently reported answer (43%, 107/246). When asked about the intensity of exercise that women believed to be safe during pregnancy, most women reported low- and moderate-intensity exercise to be safe (73%, 180/246; 83%, 205/246). Less than 37% (92/246) believed moderate- to vigorous-intensity exercise to be safe, and even fewer (30%, 73/249) believed vigorous-intensity exercise to be safe.

### 2.4. Beliefs Surrounding the Importance of Sleep and Exercise during Pregnancy

Over half of participants reported believing sleep and exercise are very important in ensuring a healthy pregnancy and birth. Moreover, many participants felt participating in more exercise would improve their sleep. Please see Table 2 below for further details.

### 2.5. Barriers to Exercise during Pregnancy

Seventy-four percent (182/247) of participants reported experiencing physical symptoms of fatigue, nausea, back pain, shortness of breath, body soreness, heartburn, ligament pain, leg cramps, restless leg syndrome, and other symptoms related to pregnancy negatively impact their ability to exercise. When asked their reasoning for not exercising, only forty-five women (10%) chose to answer. Of those that responded, the following reasons for not exercising were reported: too tired (82%), felt too unwell (58%), too busy (47%), exercise is too uncomfortable (40%), unsure what types of exercise are safe (9%), other (99%) (threatened miscarriage or pregnancy-related medical condition), dislike exercise (7%), and exercise is not safe (2%).

### 2.6. Barriers to Sleep during Pregnancy

**Figure 1:**
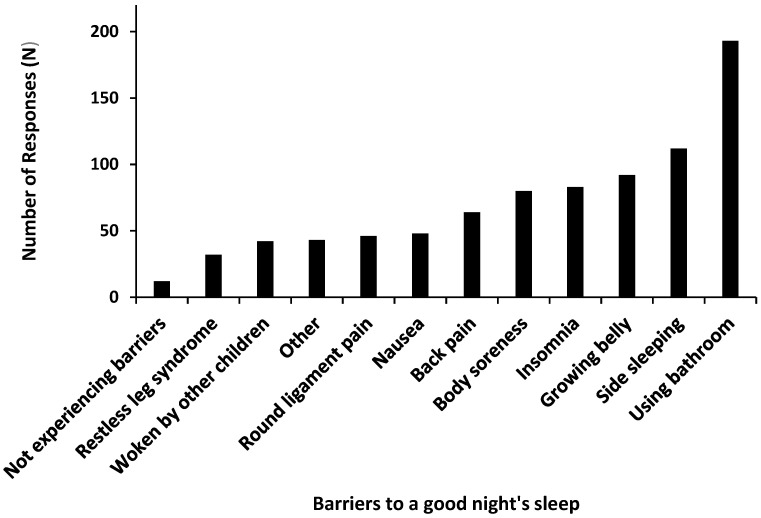
Participants’ reported barriers to sleep in their current pregnancy.

Almost all (95%, 234/246) participants reported experiencing barriers to sleep in their current pregnancy (Figure 1). Each participant was able to select more than one barrier to achieving a good night’s sleep.

### 2.7. Attitudes and Beliefs towards Sleep Disturbance by Trimester

The Kruskal–Wallis test revealed a significant difference in the number of reported sleep disturbances between trimesters such that there were less sleep disturbances reported in the first trimester than the second and third trimesters (*p* < 0.001). Post-hoc comparisons adjusted by the Bonferroni correction for multiple tests revealed significant differences between the first and second trimesters (*p* = 0.006), the first and third trimesters (*p* = 0.000), and the second and third trimesters (*p* = 0.047).

## 3. Discussion

This study had two main aims: (1) to explore the attitudes and beliefs towards sleep and exercise during pregnancy, and (2) to explore the barriers women face to achieving good sleep and engaging in appropriate levels of exercise during pregnancy. The present findings show that 98% of participants believe participating in exercise during pregnancy is safe. Sixty-five percent of participants believed exercise during pregnancy would improve sleep quality. Interestingly, 95% of participants reported waking up to use the bathroom and pain as the main barriers towards obtaining a good night’s sleep during pregnancy. Over 70% of participants reported being too tired or unwell as the main barriers to exercise during pregnancy.

### 3.1. Attitudes and Beliefs towards Sleep and Exercise

Most pregnant women within the present study believe sleep is important for a healthy pregnancy and birth. Despite this, they reported poorer sleep and frequent tiredness since becoming pregnant. These data show that pregnant women want to sleep well; however, they struggle to do so due to a range of physical and psychological barriers discussed in detail below. Participants reported a wide range of responses when asked how much sleep they believe they should be getting during pregnancy. The variance in responses may reflect the ambiguity as to how much sleep they need during pregnancy [26]. The general adult recommendations indicate that adults should obtain between 7–9 h of sleep per night [1]. However, such recommendations for pregnant women are not available [1,27].

Consistent with existing research regarding women’s attitudes and beliefs towards exercise during pregnancy, this study found that almost all participants believe participating in exercise during pregnancy is safe [19,21,28]. A growing focus by both private and government organisations to educate pregnant women and adults in general on the safety and benefits of exercise has likely been successful in providing accurate education surrounding safety [18,29]. However, women’s responses varied regarding the frequency, intensity, and duration of exercise they believe to be safe in comparison with Australian guidelines for physical activity during pregnancy [18]. Whilst pregnant women have a general belief that exercise during pregnancy is safe, knowledge regarding the specific characteristics to guide exercise during pregnancy is less established. For example, the Australian guidelines for physical activity during pregnancy recommend pregnant women engage in an accumulation of 150–300 min of moderate- to vigorous-activity per week [18]. Contrary to guideline recommendations, less than 37% of participants believed moderate- to vigorous-intensity exercise to be safe and even fewer (30%) believed vigorous-intensity exercise to be safe. The present findings are consistent with other research which found pregnant women have varying beliefs regarding the safety of moderate-to-vigorous, and vigorous exercise during pregnancy despite the well-established safety of moderate-to-vigorous and vigorous exercise [18,22]. The findings of this study suggest that some pregnant women lack knowledge surrounding the specific characteristics of exercise which are safe and recommended during pregnancy. A lack of knowledge surrounding safe exercise characteristics may offer partial explanation as to why a large number of pregnant women are not meeting recommended exercise levels despite their belief that exercise during pregnancy is generally safe [19,21,28]. This lack of knowledge surrounding safe exercise characteristics may be due in part to limited or incorrect information from medical professionals [30], not being aware of guidelines, or intrapersonal barriers to participating in exercise [21]. A study by McGee and colleagues (2018) found that obstetricians were providing incorrect information regarding resistance training, maximum heart rate during exercise, and third-trimester exercise. Furthermore, McGee and colleagues (2018) found that obstetricians were not advising their sedentary pregnant patients to exercise, despite the established safety and benefits associated with exercise for previously sedentary pregnant women.

Most participants reported believing that exercise during pregnancy is beneficial to ensuring a healthy pregnancy and birth. Interestingly, despite the perceived safety and benefits of exercise, 191 participants declined to disclose whether they are exercising in their current pregnancy. Of the 67 that did answer, 47 answered yes, and 20 answered no. This suggests disclosing whether they are exercising in their current pregnancy may be an emotionally sensitive topic for participants. Potential psychological factors which surround exercise during pregnancy such as guilt and anxiety need to be considered [31,32]. Addressing psychological factors may increase women’s exercise participation, or at the very least, increase women’s openness to exercise-related information during pregnancy.

### 3.2. Pregnant Women’s Beliefs Surrounding the Use of Exercise to Improve Sleep

Consistent with previous research surrounding beliefs towards exercise and sleep in the general population, most participants felt exercising has a positive impact on sleep quality [33]. Interestingly, only two percent of participants believe participating in more exercise would make their sleep quality worse. This suggests that many pregnant women believe exercising would improve their sleep and do not believe that increased exercise would negatively affect their sleep. Whilst the use of exercise to improve sleep during pregnancy seems like a simple fix, a large number of women are not meeting recommended levels of exercise for a variety of intrapersonal reasons [21]. The challenge may lie in determining how best to increase pregnant women’s exercise levels in a way that women find achievable and maintainable.

### 3.3. Barriers to Achieving Good Sleep and Engaging in Healthy Levels of Exercise

Overcoming barriers should be a priority for any intervention aiming to improve sleep or increase exercise levels in pregnant populations. Almost all (95%) participants reported experiencing barriers to a good night’s sleep in their current pregnancy (see Figure 1). Barriers to a good night’s sleep included getting up to use the bathroom, discomfort caused by side sleeping, their growing belly, insomnia, body soreness, back pain, nausea, round ligament pain, being woken by other children, restless leg syndrome, and other (unlisted) factors. Depression, anxiety, and other psychological factors also have the potential to influence sleep during pregnancy [8]. Stressors such as worrying about birth, the health of their baby, and motherhood negatively impact pregnant women’s sleep [34]. Likewise, hormonal changes including the production of human chorionic gonadotropin and increased progesterone cause physical changes which can result in nausea and slowed digestion in many women and disrupt sleep [34]. Exercise has been found to reduce both physical and psychological discomforts in pregnancy [17] and therefore may address many of the barriers women face to achieving a good night’s sleep.

Most participants reported experiencing uncomfortable physical symptoms related to pregnancy that negatively impact their ability to exercise. It is likely that a lack of understanding of the frequency, intensity, and duration of exercise that is safe during pregnancy, and physical symptoms such as fatigue, nausea, and a growing belly prevent women from engaging in recommended amounts of exercise [21]. Other barriers such as lack of time, lack of support, and family caregiving responsibilities are also likely to affect women’s ability to exercise [21]. The key to using exercise as a tool to manage poor sleep during pregnancy may be to determine and implement the most physically comfortable, least complicated, and least time-consuming forms of exercise that are proven effective for improving sleep.

Whilst exercise certainly cannot compensate for the effects that a growing belly has on sleep, there is evidence to suggest that exercise may reduce pain and discomfort that is commonly experienced in pregnancy. Kamali et al. (2009) and Mohamed et al. (2020) found that engaging in sitting pelvic tilt exercises reduces lower back pain in pregnant women [35,36]. Furthermore, Kamali et al. (2009) found that engaging in sitting pelvic tilt exercises during pregnancy also improved sleep [35]. Thus, it is suggested that engaging in exercise during pregnancy may improve both pain levels and sleep among pregnant women [35]. However, the direction of the causal relationship between sleep, pain, and exercise during pregnancy is still unclear.

### 3.4. Implications

The use of exercise to improve sleep during pregnancy has the potential to improve health outcomes which serve to benefit mothers and babies beyond simply feeling less tired. For example, poor sleep and exercise levels are both associated with negative health outcomes for pregnant women including increased risk of birth interventions such as caesarean section [37,38]. Evidence suggests engaging in recommended levels of exercise reduces the risk of caesarean birth [37,38]. Furthermore, adequate sleep during pregnancy has been identified as a protective factor against caesarean birth [39]. The World Health Organization (WHO) recommends that no more than 10–15% of births should result in caesarean section across a population, whilst Australia reports that on average 30% of all births in Australia are performed by caesarean section [40] with some Australian hospitals exceeding 55% [41]. The WHO notes that there is no decrease in infant mortality rates across a population when caesarean births exceed 10% and that unnecessary caesarean births can be harmful to mother and baby. Therefore, finding safe, non-pharmacological methods for improving birth outcomes for mothers and babies may be extremely beneficial. Furthermore, adequate exercise and sleep during pregnancy is associated with decreased risk of gestational diabetes and improved postpartum mental health [37,42]. Thus, the benefits of increasing exercise levels during pregnancy apply not only to sleep, but to other important health outcomes as well.

### 3.5. Limitations

Some limitations need to be acknowledged. One of the major limitations of this study is the absence of a control group of non-pregnant women. Future studies may wish to include a control group to build on the present findings. The generalisability of these findings may be limited as the majority of participants had completed education beyond year 12 level and had average household incomes exceeding $100,000. Such results are not generalisable to areas where education and income averages fall below these levels. While the sample size obtained in this study (258 participants) is adequate in comparison to similar cross-sectional studies among pregnant women [23,43,44], it fell short of our sample size calculation which recommended approximately 384 participants. Furthermore, potential bias is introduced by the reduced number of subjects answering some of the questions. In order to respect the rights of participants, they were allowed to skip questions they did not feel comfortable answering. Lastly, it was beyond the scope of this survey to investigate the effect psychological factors such as depression, stress, and anxiety may have on participants’ ability to sleep or engage in exercise during pregnancy. Investigating the effect of psychological factors on sleep and exercise during pregnancy may be a worthwhile direction for future research.

### 3.6. Future Directions

The findings from the present study highlight the need for a better understanding of women’s sleep experiences during pregnancy, and demonstrate how exercise may improve sleep and health outcomes. A small number of studies have investigated the use of exercise to manage poor sleep during pregnancy [25]. Of these, findings suggest sleep is improved by exercise, but further research is needed to determine the frequency, intensity, duration, and type of exercise most beneficial to sleep during pregnancy [25]. One study suggests water exercise may be effective at improving sleep during pregnancy [45]. Walking [46] and yoga [47] have proven effective in non-pregnant populations at improving sleep and may be worthwhile directions for future investigation in pregnant populations.

## 4. Materials and Methods

### 4.1. Participants

To be included in the study, participants were required to be over 16 years of age and residing in Australia. Participants were excluded if they were not currently pregnant. Participants were drawn from a convenience sample recruited via social media platform Facebook (Menlo Park, CA, USA), between January and June 2019. Pregnant women were invited to complete a 51-question survey investigating their attitudes and beliefs towards sleep and exercise in pregnancy. The survey took approximately 10 to 15 min to complete. Participation was voluntary, and participants were able withdraw at any time. A sample size calculation with finite population was conducted considering the following assumptions: standard normal distribution with a confidence interval of 95% (Z = 1.96), absolute precision or tolerable margin of error (d = 0.05), and the population of pregnant women in Australia based on the most recent statistics obtained in 2020 (n = 291,712).

### 4.2. Procedure and Materials

A cross-sectional, within-subjects design was employed. Participants accessed the online survey via SurveyMonkey (Momentive, San Mateo, CA, USA) [48]. An information sheet was presented, and informed consent was requested prior to participants progressing with the survey. The survey included 51 questions examining participants’ demographics, pregnancy information (e.g., gestational age), and current sleep- and exercise-related attitudes, beliefs, and barriers. The survey included a 5-point Likert scale, open- and closed-ended, and multiple-choice questions. Exercise-related questions were derived from the validated Exercise Safety Beliefs Questionnaire and adapted to suit the current study [49]. The remaining survey questions were developed specifically to answer the present research questions regarding attitudes, beliefs, and barriers towards sleep and exercise during pregnancy where no validated measures were available. The survey was pilot tested among a sample of 10 female participants who have all previously been pregnant. This study was granted ethical approval by CQUniversity’s Human Research Ethics Committee (Ethics approval number 0000021377). Not all survey responses are presented in the present paper due to the large amount of data collected.

### 4.3. Statistical Analyses

All statistical analyses were computed using SPSS Statistics (v25, IBM, Armonk, NY, USA). Descriptive statistics were conducted to explore means, standard deviations, and percentages. In circumstances where the Kolmogorov–Smirnov test of normality was violated, the differences in the number of sleep disturbances between trimesters of pregnancy were calculated using the non-parametric alternative Kruskal–Wallis test. Post-hoc pairwise comparisons adjusted by the Bonferroni correction for multiple tests were performed. Statistical significance was determined with alpha set as <0.05.

## 5. Conclusions

Given the importance of achieving healthy levels of sleep and exercise during pregnancy, this study aimed to (1) explore the attitudes and beliefs towards sleep and exercise during pregnancy, and (2) explore the barriers women face to achieving good sleep and engaging in appropriate levels of exercise during pregnancy. Findings show that almost all participants believed participating in exercise during pregnancy is safe, and more than half believed exercise during pregnancy would improve sleep quality. Participants reported many intrapersonal barriers to obtaining a good night’s sleep and exercising adequately during pregnancy. Overcoming barriers should be a priority for any intervention aiming to improve sleep or increase exercise participation in pregnant populations. The findings from the present study highlight the need for a better understanding of women’s sleep experiences during pregnancy, and demonstrate how exercise may improve sleep and health outcomes.

## Figures and Tables

**Table 1:** Participant demographic characteristics.

		n	%
Age	17–30	98	37.98
	31–42	160	62.01
Trimester	First	46	17.89
	Second	109	42.41
	Third	95	36.96
Parity	Primiparous	187	72.48
	Multiparous	71	27.52
Marital status	Married	171	66.28
	Partnered	82	31.78
	Single	5	1.94
Education	Less than year 10	1	0.39
	Year 10 or equivalent	6	2.33
	Year 12 or equivalent	24	9.30
	TAFE/VET Qualification	33	12.79
	Bachelor degree	114	44.19
	Postgraduate degree	80	31.01
Employment status	Employed full-time	154	59.69
	Employed part-time	61	23.64
	Currently not employed	21	8.14
	Full-time carer of children	22	8.53
Annual household income	<$30,000	9	3.49
	$30,001–$50,000	12	4.65
	$50,001–$70,000	14	5.43
	$70,001–$100,000	31	12.02
	$100,001–$120,000	41	15.89
	$120,001–$150,000	40	15.50
	>$150,000	94	36.43
	Unsure/prefer not to say	17	6.59

Data listed in Table 1 represent participants within the study.

**Table 2:** Beliefs surrounding the importance of sleep and exercise during pregnancy.

Question: How important do you believe sleep is in ensuring you have a healthy pregnancy and birth?	Response	n (242)	%
	Very important	188	77.69%
	Somewhat important	49	20.25%
	Neutral	4	1.65%
	Not very important	0	0.00%
	Not important at all	1	0.41%
Question: How important do you believe exercise is in ensuring you have a healthy pregnancy and birth?	Response	n (65)	%
	Very important	38	58.46%
	Somewhat important	21	32.31%
	Neutral	6	9.23%
	Not very important	0	0.00%
	Not important at all	0	0.00%
Question: Do you believe participating in more exercise during pregnancy would:	Response	n (244)	%
	Improve the quality of your sleep	164	67.21%
	Make the quality of your sleep worse	4	1.64%
	Have no difference	76	31.15%

## Data Availability

The datasets analyzed in this study are available from the corresponding author upon reasonable request.
